# Baseline platelet count and long-term clinical outcomes in patients with acute venous thromboembolism: a prospective cohort study

**DOI:** 10.1007/s00277-024-05982-8

**Published:** 2024-09-09

**Authors:** Johann Stuby, Odile Stalder, Andreas Limacher, Marc Righini, Nicolas Rodondi, Tobias Tritschler, Marie Méan, Drahomir Aujesky

**Affiliations:** 1 Department of General Internal Medicine, Inselspital, Bern University Hospital, University of Bern, Bern, Switzerland; 2 Department of Clinical Research, CTU Bern, University of Bern, Bern, Switzerland; 3 Department of Angiology and Hemostasis, Geneva University Hospital, Geneva, Switzerland; 4 Institute of Primary Health Care (BIHAM), University of Bern, Bern, Switzerland; 5 Department of Internal Medicine, Lausanne University Hospital, Lausanne, Switzerland

**Keywords:** Bleeding, Mortality, Platelets, Recurrence, Venous thromboembolism

## Abstract

An abnormal platelet count (PC) is common in acute venous thromboembolism (VTE) but its relationship with clinical outcomes remains ill-defined. We aimed to explore the association between baseline PC and the long-term risk of clinically relevant outcomes in a prospective cohort of 991 patients with acute VTE. We classified patients into four PC groups: very low (< 100 G/l), low (≥ 100 to < 150 G/l), normal (≥ 150 G/l to ≤ 450 G/l), and high (> 450 G/l). The primary outcome was major bleeding (MB), secondary outcomes were recurrent VTE and overall mortality. We examined the association between PC and clinical outcomes, adjusting for confounders, competing risk for mortality, and periods of anticoagulation. After a median follow-up of 30 months, 132 (13%) of patients experienced MB, 122 (12%) had recurrent VTE, and 206 (21%) died. Compared to patients with a normal PC, patients with a very low PC had a sub-distribution hazard ratio (SHR) for MB of 1.23 (95% confidence interval [CI] 0.52–2.91) and those with a high PC a SHR of 1.87 (95%CI 0.82–4.29). Patients with a low PC had a twofold increased VTE recurrence risk (SHR 2.05, 95%CI 1.28–3.28). Patients with low and very low PC had a hazard ratio for mortality of 1.43 (95%CI 0.99–2.08) and of 1.55 (95%CI 0.80–2.99), respectively. Our findings do not suggest a consistent relationship between baseline PC and long-term clinical outcomes in patients with VTE.

## Introduction

Abnormal platelet counts (PC) are common in patients with venous thromboembolism (VTE), with about 4–23% of patients having thrombocytopenia [1–4] and 3–4% thrombocytosis [2, 3]. In patients with VTE, both thrombocytopenia and thrombocytosis appear to be associated with overall or fatal major bleeding (MB) [2–6]. While the relationship between thrombocytopenia and bleeding remains poorly defined [7], the link between high PC and bleeding is even less clear. Possible explanations are that a high PC may represent reactive thrombocytosis due to comorbid conditions with an increased bleeding risk (e.g., cancer) [8] or may lead to acquired von Willebrand disease [9]. An increased PC may also be associated with a decreased mean platelet volume and lower platelet reactivity [6].

As platelets are involved in the initiation and progression of venous thrombosis [10], an elevated PC could increase the risk of a first VTE or VTE recurrence. While an elevated PC was shown to increase thrombosis risk in medical inpatients [11], cancer [12, 13], and after critical illness [14], a meta-analysis of retrospective studies, excluding patients with cancer and other comorbid diseases, found no relationship between PC and a first VTE [15]. In a registry study with long-term follow-up, a low PC was not associated with recurrent VTE [4]. While one study showed an association between a low PC and an increased risk of overall mortality following VTE [4], another did not [1].

The few studies examining the relationship between PC and clinical outcomes among patients with VTE were limited by exclusion of patients with a PC < 100 G/l [16], focus on thrombocytopenia without consideration of thrombocytosis [1, 4–6], failure to account for competing risk [1–5], or a short follow-up of 3 months only [1, 2, 5, 16]. We therefore aimed to examine the association between baseline PC and long-term clinical outcomes in a prospective Swiss multicenter study of older patients with acute VTE.

## Methods

### Study population

We used data from the SWiss venous Thromboembolism COhort study 65 + (SWITCO65 +), a prospective multicenter cohort study, which enrolled and followed-up in- and outpatients aged ≥ 65 years with objectively diagnosed VTE from nine Swiss university and non-university hospitals between September 2009 and December 2013. Patients with catheter-associated thrombosis, thrombosis at a different site than the lower limb, conditions incompatible with follow-up (e.g., terminal illness with life expectancy < 3 months), inability to provide informed consent (e.g., due to severe dementia), or insufficient proficiency in German or French were not included in the study. A detailed description of the study methods of SWITCO65 + was published elsewhere [17].

### Patient data collection

Trained study personnel prospectively collected information about baseline patient demographics (age, sex), localization of the index VTE (PE ± DVT, DVT only), medical history (prior MB or VTE, active cancer, chronic renal disease, physical activity level), systolic blood pressure, laboratory findings (hemoglobin), concomitant treatment with platelet inhibitors, and VTE-related treatments (anticoagulants, thrombolysis) using standardized data collection forms. In patients receiving vitamin K antagonists (VKAs), we also collected all international normalized ratio (INR) values.

The PC at the time of VTE diagnosis was abstracted from each patient’s medical chart. As thrombocytopenia is commonly defined as PC of < 150 G/l [18] and thrombocytosis as a PC of > 450 G/l [19], we categorized patients into four groups based on their PC: very low (< 100 G/l), low (≥ 100 to < 150 G/l), normal (≥ 150 G/l to ≤ 450 G/l), and high (> 450 G/l).

### Study outcomes

The primary outcome was MB during follow-up. MB was defined as fatal bleeding, bleeding at critical sites (i.e., intracranial, intraocular, pericardial, retroperitoneal, intraspinal, intraarticular, or intramuscular with compartment syndrome), or bleeding leading to a reduction of hemoglobin of ≥ 20 g/l or resulting in transfusion of ≥ 2 units of packed red blood cells [20]. Secondary outcomes were recurrent VTE and overall mortality. VTE recurrence was defined as new fatal or nonfatal PE or new DVT (proximal or distal) based on previously published criteria [21].

Patients were followed-up with a surveillance face-to-face evaluation after 3 months and a telephone call after 6 months, which were then alternated semi-annually. During each contact, information about any bleeding event, VTE recurrence, and death was obtained. In case of an event, the corresponding medical charts were reviewed and the patients’ primary care physician and/or family members were interviewed. A committee of three blinded clinical experts adjudicated the outcomes. Death was considered bleeding-related if it followed an intracranial hemorrhage or a bleeding event resulting in hemodynamic instability. PE-related death was defined as either definite fatal PE (proven by autopsy or clinically severe PE in the absence of an alternative diagnosis) or a possible fatal PE (sudden death without any apparent cause) [22]. Final classification was based on the full consensus of this committee.

### Statistical analysis

We compared patient baseline characteristics by PC group using the chi-squared test for categorical and the Kruskal–Wallis test for continuous variables. In patients receiving VKAs, we also assessed the quality of anticoagulation, expressed as the percentage of time spent in the therapeutic range of the INR of 2.0–3.0 based on the Rosendaal method [23].

We used the Kaplan–Meier method and the log-rank test to compare the cumulative 3- and 36-month incidences of MB, recurrent VTE, and overall mortality by PC. Because the association between PC and clinical outcomes may not be linear, we graphically visualized the unadjusted hazard ratio (HR) for MB, recurrent VTE, and overall mortality relative to the mean PC as a continuous function of the PC using fractional polynomial models, with automatic selection of the best fitting power function (e.g., linear, quadratic, or higher power).

We explored the association between PC and the time to a first MB over the entire follow-up period using competing risk regression according to Fine and Gray [24], accounting for non-bleeding-related death as a competing event. We adjusted the model for known predictors of MB, i.e., age, sex, active cancer, chronic renal disease, low physical activity level, anemia, concomitant platelet inhibitor therapy, and periods of anticoagulation as a time-varying covariate [4, 25, 26]. The strength of the association was expressed as a sub-distribution hazard ratio (SHR) with corresponding 95% confidence intervals (CI). We also used competing risk regression to explore the association between PC and the time to a first VTE recurrence, accounting for non-VTE-related death as a competing event. The model was adjusted for previously described predictors of VTE recurrence, i.e., age, sex, history of VTE, active cancer, and periods of anticoagulation as a time-varying covariate [4, 27, 28]. Finally, we assessed the association between PC and the time to death from all causes using Cox regression analysis, adjusting for known mortality predictors in VTE, including age, sex, active cancer, low physical activity, systolic blood pressure of < 100 mm Hg, and periods of anticoagulation as a time-varying covariate [29]. As cancer is associated with both thrombocytopenia and thrombocytosis and is an important risk factor for MB, recurrent VTE, and death in VTE [12, 30–32], we also explored the association between PC and clinical outcomes in the subgroup of patients with active cancer using the same models. Due to the limited number of thrombocytopenic cancer patients, we merged the groups with very low PC and low PC into a single group.

To address missing data for PC and other covariates, we used multiple imputation by chained equations, utilizing baseline patient characteristics. Predictive mean matching and logit models were used to impute non-binary and binary variables, respectively. In total, 50 imputed data sets were generated, which we analyzed using Rubin’s rules to combine results across data sets [33]. A *P*-value of < 0.05 was considered statistically significant. All analyses were performed using Stata version 17 (Stata Corporation, College Station, Texas, United States).

## Results

### Study sample

Of 1003 patients enrolled in SWITCO65 + , we excluded 12 who did not allow the use of their data or withdrew from the study within 1 day of enrollment, leaving a final study sample of 991 patients. Analyzed patients had a median age of 75 years (interquartile range [IQR] 69–81), and 463 (47%) were women. Overall, 35 patients (3.5%) had a very low (< 100 G/l), 105 (11%) a low (≥ 100 G/l to < 150 G/l), 759 (77%) a normal (≥ 150 G/l to ≤ 450 G/l), and 29 (3%) a high PC (> 450 G/l). Only 13 patients (1%) had a PC of < 50 G/l. Patients with a very low or high PC were more likely to have a history of MB and anemia and patients with a higher PC more often received platelet inhibitors than those with a low/normal PC (Table 1). The proportion of patients who received initial therapeutic parenteral anticoagulation did not vary by PC.

**Table 1:** Patient baseline characteristics by platelet count

Characteristic	< 100 G/l (*n* = 35)	≥ 100 to < 150 G/l (*n* = 105)	≥ 150 to ≤ 450 G/l (*n* = 759)	> 450 G/l (*n* = 29)	*P*-value
	n (%) or median (interquartile range)				
Age	75 (71–78)	74 (68–82)	75 (69–81)	74 (69–77)	0.51
Male	16 (46)	64 (61)	401 (53)	12 (41)	0.17
Localization of VTE					0.85
PE ± DVT	24 (69)	76 (72)	556 (73)	20 (69)	
DVT only	11 (31)	29 (28)	203 (27)	9 (31)	
History of MB	8 (23)	14 (13)	73 (10)	5 (17)	0.04
History of VTE	12 (34)	36 (34)	208 (27)	5 (17)	0.21
Active cancer^1^	11 (31)	24 (23)	135 (18)	6 (21)	0.14
Chronic renal disease^2^	10 (29)	22 (21)	148 (19)	3 (10)	0.33
Low physical activity^3^	16 (46)	45 (43)	284 (37)	5 (17)	0.06
SBP < 100 mm Hg	0 (0)	8 (8)	25 (3)	1 (3)	0.12
Anemia^4^	23 (66)	45 (43)	293 (39)	27 (93)	< 0.001
Platelet inhibitors^5^	6 (17)	35 (33)	297 (39)	15 (52)	0.014
Initial parenteral AC					0.81
LMWH	16 (46)	48 (46)	345 (45)	13 (45)	
UFH	15 (43)	38 (36)	260 (34)	11 (38)	
Others	2 (6)	18 (17)	127 (17)	3 (10)	
None	2 (6)	1 (1)	27 (4)	2 (7)	
VKA therapy	27 (77)	90 (86)	662 (87)	24 (83)	0.29
Thrombolysis^6^	2 (6)	6 (6)	22 (3)	0 (0)	0.22

Data were missing for platelet count (n = 63 [6%]), history of MB (n = 1 [< 1%]), low physical activity (n = 3 [< 1%]), SBP < 100 mm Hg (n = 18 [2%]), and hemoglobin (n = 63 [6%])

*AC*, anticoagulation; *DVT*, deep vein thrombosis; *LMWH*, low-molecular-weight heparin; *MB*, major bleeding; *PE*, pulmonary embolism; *SBP*, systolic blood pressure; *UFH*, unfractionated heparin; *VKA*, vitamin K antagonist; *VTE*, venous thromboembolism

^1^Cancer requiring surgery, chemotherapy, radiotherapy, or palliative care during the last 3 months

^2^Chronic renal failure requiring hemodialysis or not (diabetic or hypertensive nephropathy, chronic glomerulonephritis or interstitial nephritis, myeloma-related nephropathy, or cystic kidney disease)

^3^Mostly lying/sitting activity, or avoidance to climb stairs, or carry light weight < 5 kg

^4^Serum hemoglobin < 13 g/dl for men or < 12 g/dl for women

^5^Aspirin, clopidogrel, ticagrelor, prasugrel, or nonsteroidal anti-inflammatory drug

^6^Systemic or catheter-related thrombolysis

The median follow-up duration was 30 months (IQR 19–36 months), and the median duration of initial therapeutic anticoagulation was 8 months (IQR 4–24 months). Among the 780 patients (79%) who received VKAs and who had available INR and PC values, the percentage of time spent within the therapeutic INR range did not differ by PC and was 63% for patients with a very low or low PC, 64% for patients with a normal PC, and 65% for those with a high PC (*P* = 0.79).

### Major bleeding

**Fig. 1.**
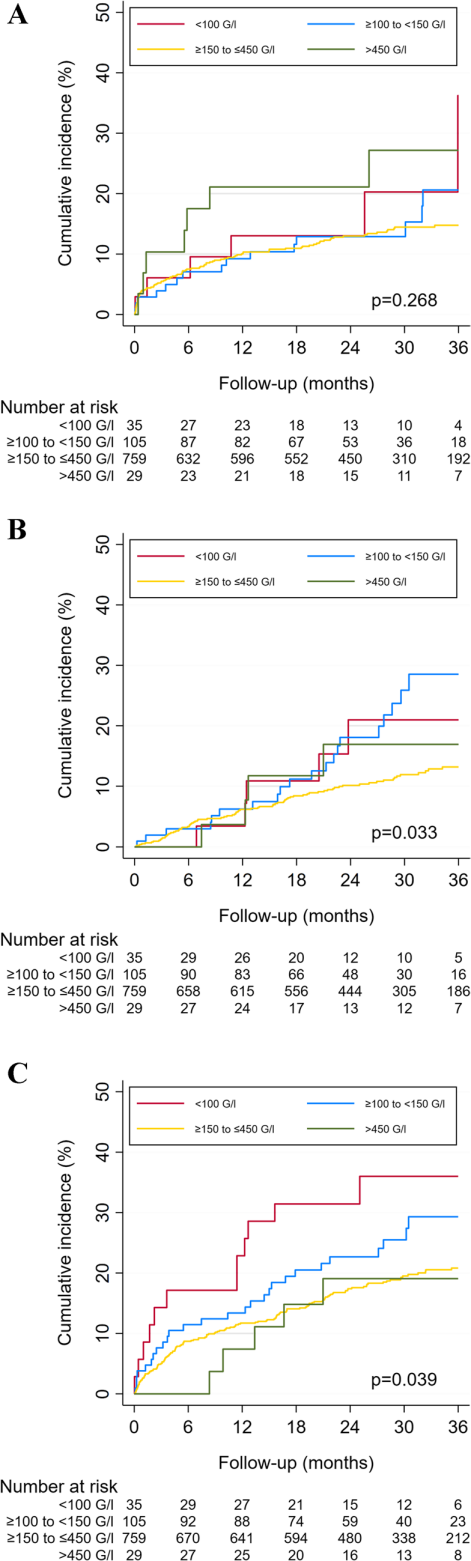
36-month cumulative incidence of clinical outcomes by platelet count. Panel **A**. Major bleeding. The cumulative incidence was 36% (95% confidence interval [CI] 14–74%) for patients with a very low PC, 21% (95%CI 12–33%) for patients with a low PC, 15% (95%CI 12–18%) for patients with a normal PC, and 27% (95%CI 14–50%) for those with a high PC (*P* = 0.268 by the log-rank test). Panel **B**. Recurrent venous thromboembolism. The cumulative incidence was 21% (95% confidence interval [CI] 9–44%) for patients with a very low PC, 29% (95%CI 19–42%) for patients with a low PC, 13% for patients with a normal PC (95%CI 11–16%), and 17% (95%CI 7–40%) for those with a high PC (*P* = 0.033 by the log-rank test). Panel **C**. Overall mortality. The cumulative incidence was 36% (95% confidence interval [CI] 22–55%) for patients with a very low PC, 29% (95%CI 21–40%) for patients with a low PC, 21% (95%CI 18–24%) for patients with a normal PC, and 19% (95%CI 8–40%) for those with a high PC (*P* = 0.039 by the log-rank test)

**Fig. 2:**
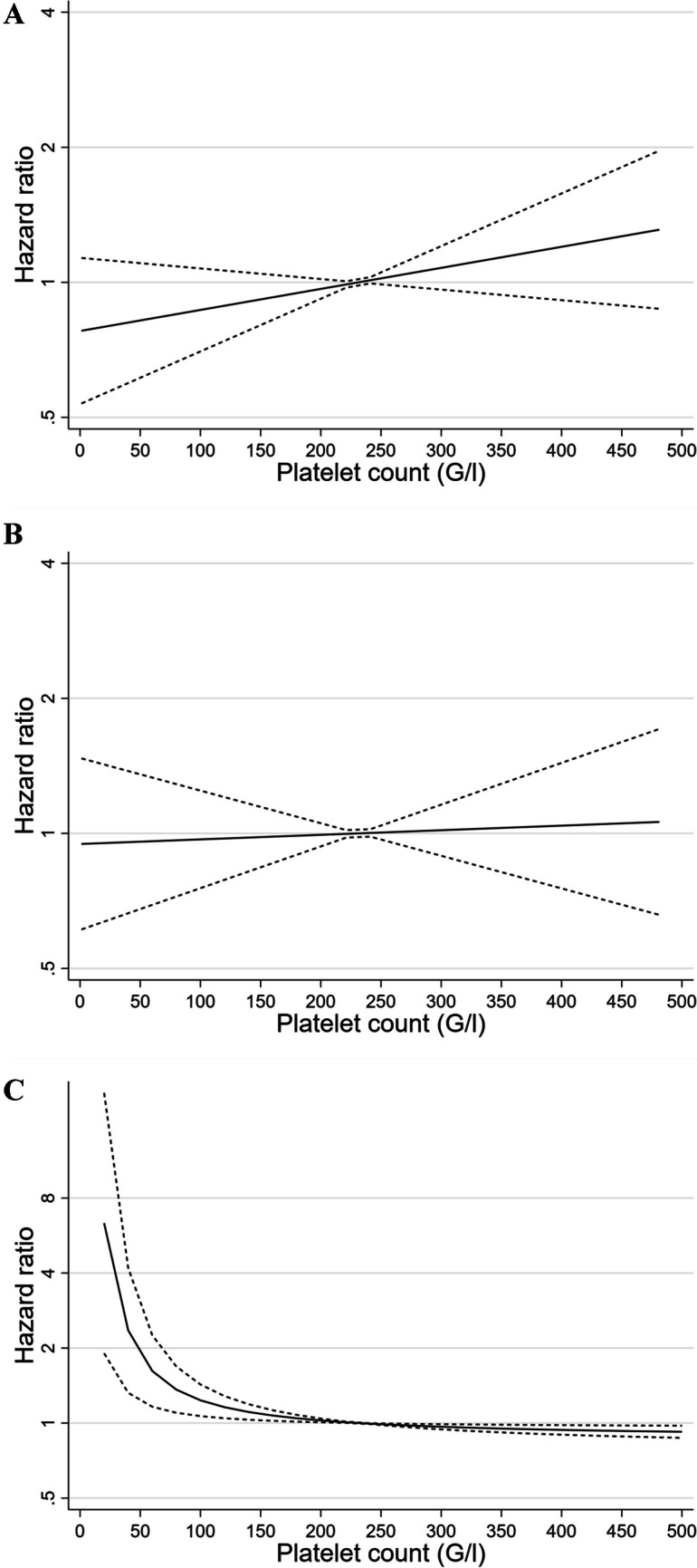
Hazard ratio of clinical outcomes as a continuous function of platelet count. Panel **A**. Major Bleeding. The solid line indicates the hazard ratio of major bleeding on a log-transformed y-axis relative to the mean platelet count of 231 G/l. The dashed lines represent the 95% confidence intervals. Panel **B**. Recurrent venous thromboembolism. The solid line indicates the hazard ratio of recurrent venous thromboembolism on a log-transformed y-axis relative to the mean platelet count of 231 G/l. The dashed lines represent the 95% confidence intervals. Panel **C**. Overall mortality. The solid line indicates the hazard ratio of death on a log-transformed y-axis relative to the mean platelet count of 231 G/l. The dashed lines represent the 95% confidence intervals

Overall, 132 (13%) of patients suffered MB during follow-up. The 3-month cumulative incidence of MB was similar across PC groups and was 6% (95%CI 2–22%), 4% (95%CI 1–10%), 5% (95%CI 4–7%), and 10% (95%CI 3–29%) in patients with very low, low, normal, and high PC, respectively (*P* = 0.623 by the log-rank test). The 36-month cumulative incidence of MB did not differ by PC (Fig. 1Panel A). The hazard of MB as a continuous function of PC slightly increased with increasing PC during follow-up (Fig. 2Panel A). Although patients with a very low and a high PC had a somewhat increased adjusted risk of MB, the association was not statistically significant, neither in the full sample nor in the subgroup of patients with cancer (Table 2).

**Table 2:** Association between platelet count and clinical outcomes

	Full sample (*n* = 991)			Patients with cancer (*n* = 178)^1^		
Outcome	Number	Adjusted SHR^2^ (95%CI)	*P*-value	Number	Adjusted SHR^5^ (95%CI)	*P*-value
Major bleeding						
< 100 G/l	6	1.23 (0.52–2.91)	0.636	5	0.81 (0.29–2.28)	0.692
≥ 100 G/l to < 150 G/l	16	1.12 (0.66–1.91)	0.679			
≥ 150 G/l to ≤ 450 G/l	102	Reference		23	Reference	
> 450 G/l	7	1.88 (0.82–4.29)	0.136	2	1.6 (0.32–8.21)	0.567
		**Adjusted SHR**^**3**^ **(95%CI)**	***P*-value**		**Adjusted SHR**^**5**^ **(95%CI)**	***P*-value**
VTE recurrence						
< 100 G/l	5	1.19 (0.48–2.98)	0.704	7	2.83 (0.99–8.11)	0.053
≥ 100 G/l to < 150 G/l	23	2.05 (1.28–3.28)	0.003			
≥ 150 G/l to ≤ 450 G/l	90	Reference		9	Reference	
> 450 G/l	4	1.18 (0.41–3.37)	0.757	1	2.78 (0.34–22.9)	0.342
	**Number**	**Adjusted HR**^**4**^ **(95% CI)**	***P*-value**	**Number**	**Adjusted HR**^**5**^ **(95% CI)**	***P*-value**
Overall mortality						
< 100 G/l	12	1.55 (0.80–2.99)	0.190	25	1.76 (1.09–2.83)	0.020
≥ 100 G/l to < 150 G/l	31	1.43 (0.99–2.08)	0.058			
≥ 150 G/l to ≤ 450 G/l	158	Reference		67	Reference	
> 450 G/l	5	0.81 (0.28–2.37)	0.703	3	0.81 (0.20–3.26)	0.767

*CI*, confidence interval; *HR*, hazard ratio; *SHR*, sub-distribution hazard ratio; *VTE*, venous thromboembolism

^1^Patients with a platelet count of < 100 G/l and those with platelet count of ≥ 100 G/l to < 150 G/l were merged into a single group (< 150 G/l). Models for cancer were not adjusted for active cancer

^2^Adjusted for age, sex, active cancer, chronic renal disease, low physical activity, anemia, concomitant antiplatelet therapy, and periods of anticoagulation

^3^Adjusted for age, sex, history of VTE, active cancer, and periods of anticoagulation

^4^Adjusted for age, sex, active cancer, low physical activity, systolic blood pressure of < 100 mm Hg, and periods of anticoagulation

^5^Models were adjusted for the same co-variates as in the full sample (except active cancer)

### Venous thromboembolism recurrence

Overall, 122 patients (12%) had recurrent VTE during follow-up. The 36-month cumulative incidence of recurrence differed significantly by PC and was highest in patients with a low PC (29%, 95%CI 19–42%) (Fig. 1Panel B). The hazard of recurrence as a continuous function of PC did not change across PC, indicating that there was no relationship between PC and recurrent VTE (Fig. 2Panel B). Patients with a low PC (but not those with a very low PC) had a twofold higher adjusted risk of recurrence than patients with a normal PC in the full sample (SHR 2.05, 95%CI 1.28–3.28). A similar trend was also seen in patients with cancer (SHR 2.83, 95%CI 0.99–8.11) (Table 2).

### Overall mortality

Of the 206 (21%) of patients who died during follow-up, 13 (6%) died from MB, 36 (18%) from definite or possible PE, and 70 (34%) from cancer. Among the 12 patients with a very low PC who died, none died from bleeding and 5 from cancer. Similarly, among the 25 cancer patients with a PC < 150 G/l who died, 1 died from bleeding and 16 from cancer. The 36-month cumulative incidence of death varied significantly by PC and was highest in patients with a very low PC (21%, 95%CI 18–24%) (Fig. 1Panel C). The hazard of death as a continuous function of PC was highest among patients with the lowest PC values, indicating an L-shaped relationship between PC and mortality (Fig. 2Panel C). After adjustment, mortality did not statistically significantly differ by PC in the full sample, although patients with a lower PC had a somewhat higher mortality risk. Patients with cancer who had a low PC had a higher risk of death than those with a normal PC (HR 1.76, 95%CI 1.09–2.83) (Table 2).

## Discussion

Overall, 15% of patients were thrombocytopenic and 3% had thrombocytosis at the time of presentation for acute VTE. Patients with a low PC (but not those with a very low PC) had a significantly increased risk of recurrent VTE compared to those with a normal PC. Although the risk of MB was somewhat increased in patients with a very low and particularly in those with a high PC, the differences failed to achieve statistical significance. While PC and overall mortality appeared to be (albeit statistically not significantly) inversely related in the full sample, a PC < 150 G/l was associated with an almost twofold risk of death in the subgroup of patients with active cancer only. Overall, our results did not show a straightforward association between the PC at presentation and long-term clinical outcomes in older patients with VTE.

The prevalence of thrombocytopenia and thrombocytosis in our study was consistent with prior findings from registry studies, in which 12–23% and 3–4% of patients had a PC of < 100 G/l and > 450 G/l at VTE diagnosis, respectively [2–4]. Although patients with very low and in particular those with high PC had a somewhat higher risk of MB, the PC was not independently associated with the long-term risk of MB in our prospective cohort. While two analyses from an international VTE registry (RIETE) showed a 2–4 times increased risk of 3-month overall and fatal MB in patients with a PC of < 80 to 100 G/l [2, 5], a prior analysis from the same registry did not [34]. In the COMMAND VTE registry [4], the only prior study examining long-term outcomes (median follow-up: 40 months) in thrombocytopenic VTE patients, those with a PC of < 100 G/l had a twofold higher MB risk than those with a PC of > 150 G/l. Although the majority of studies point to thrombocytopenia at baseline as a risk factor for bleeding in VTE, it is uncertain to which extent thrombocytopenia is associated with an increased MB risk in patients with VTE. This is also reflected by the fact that 6 out of 10 commonly cited bleeding risk scores for anticoagulated patients with VTE and atrial fibrillation do not incorporate the PC as a predictor variable [35]. Limited evidence from small retrospective studies of patients with cancer and VTE suggest that therapeutic-dose anticoagulation may be safe for patients with a PC > 50 G/l [36–38], and guidelines do not recommend a dose reduction or platelet transfusions in such patients [7, 36].

Interestingly, patients with a high PC had an almost twofold higher risk of MB than those with a normal PC in our study, although the difference was not statistically significant. Our results are consistent with prior evidence from a registry (RIETE) [2] and the Tromsø cohort study [6] in which patients with a PC of > 450 G/l and ≥ 300 G/l had a 2.1 and 3.2-fold higher risk of MB, respectively. The increased bleeding risk in patients with a higher PC may be the consequence of primary (e.g., myeloproliferative diseases) and secondary causes of thrombocytosis (e.g., cancer, iron deficiency) [2]. It has also been speculated that the presence of an increase in the PC, even within a normal range, would be associated with a lower platelet reactivity, as reflected by a decrease in mean platelet volume, which could predispose to anticoagulation-related bleeding [6].

We found a twofold higher risk of recurrent VTE in patients with a low PC but not in those with a very low PC or high PC. It is possible that the thrombogenic effect of comorbid conditions (e.g., cancer) was partially neutralized by a very low PC and the higher frequency of antiplatelet treatment in patients with a high PC in our study, reducing the risk of recurrence in these patient groups. In the COMMAND VTE registry [4], a PC of 100–150 G/l (but not a PC < 100 G/l) also appeared to carry a slightly increased risk of recurrent VTE (+ 30%) compared to a PC > 150 G/l, but the difference was not statistically significant. In contrast to patients with cancer in whom thrombocytosis is associated with a first VTE [12, 39, 40], there is currently little evidence for a relationship between PC and VTE recurrence.

Patients with a very low PC had an increased long-term risk of overall mortality (+ 55%) in our study, although the association achieved statistical significance in the subgroup of patients with cancer only. The fact that no patient with a very low PC in the full sample died from bleeding indicates that comorbid conditions rather than thrombocytopenia are the main cause of death in patients with a very low PC. This finding is consistent with results from the COMMAND VTE registry [4], in which a PC of < 100 G/l was associated with a 54% mortality increase compared to a PC > 150 G/l.

The strengths of our study include its prospective design, the long-term follow-up and, on an analytical level, the adjustment for competing risks. However, our study has also potential limitations. First, our analysis included patients aged ≥ 65 years only, and thus our results may not be generalizable to younger patients with VTE. Second, we measured PC at baseline and had no information about the subsequent course of the PC, i.e., whether PC abnormalities were transient or persisting. It is conceivable that long-term clinical outcomes may have a stronger association with a temporally closer PC than with the baseline PC. Third, we could not assess the causes of low and elevated PC and the direct impact of these conditions on patient prognosis. Fourth, there were relatively few patients with very low and high PC and our study may be underpowered to detect statistically significant associations with outcomes in these subgroups. According to expert consensus, patients with a hematologic cancer who have acute VTE who have a PC of ≥ 50 G/l should receive full-dose anticoagulation, those with a PC of 30 to < 50 G/l half-dose anticoagulation, and those with a PC < 30 G/l prophylactic anticoagulation with additional measures (vena cava filter, platelet transfusions) [41]. Finally, like other studies examining the relationship between PC and outcomes in VTE [2, 3], our study was from the pre-direct oral anticoagulant (DOAC) era. Limited evidence suggests that DOACs may be safer and more effective than VKAs in treating VTE in older patients [42]. Thus, our results may have been different if DOACs had been used to treat VTE.

In conclusion, although a very low and high PC was related to a higher risk of MB, a low PC with an increased risk of recurrent VTE, and a very low PC with a higher mortality risk, the magnitude of these associations was rather small, which for the most part failed to achieve statistical significance. Overall, we found no consistent association between PC at baseline and long-term clinical outcomes in patients with acute VTE. Our results suggest that baseline PC may not be particularly useful in risk-stratifying patients with acute VTE.

## Data Availability

The authors confirm that the data supporting the findings of this study are available within the article.

## References

[1] Goldhaber SZ, Visani L, De Rosa M (1999) Acute pulmonary embolism: clinical outcomes in the International Cooperative Pulmonary Embolism Registry (ICOPER). The Lancet 353:1386–1389. 10.1016/S0140-6736(98)07534-5 10.1016/s0140-6736(98)07534-5 10227218

[2] Di Micco P, Ruiz-Giménez N, Nieto JA, Aujesky D, del Molino F, Valle R, Barrón M, Maestre A, Monreal M, investigators R (2013) Platelet count and outcome in patients with acute venous thromboembolism. Thromb Haemost 110:1025–34 23925476 10.1160/TH13-04-0352

[3] Giorgi-Pierfranceschi M, Di Micco P, Cattabiani C, Guida A, Pagán B, del Valle Morales M, Salgado E, Surinach JM, Tolosa C, Monreal M (2015) Platelet count and major bleeding in patients receiving vitamin K antagonists for acute venous thromboembolism, findings from real world clinical practice. Medicine 94(47):e1915 26632687 10.1097/MD.0000000000001915 PMC5058956

[4] Yamashita Y, Morimoto T, Amano H, Takase T, Hiramori S, Kim K, Oi M, Akao M, Kobayashi Y, Toyofuku M (2018) Influence of baseline platelet count on outcomes in patients with venous thromboembolism (from the COMMAND VTE Registry). Am J Cardiol 122:2131–2141 30293653 10.1016/j.amjcard.2018.08.053

[5] Nieto J, Solano R, Ruiz-Ribo M, Ruiz-Gimenez N, Prandoni P, Kearon C, Monreal M, Investigators R (2010) Fatal bleeding in patients receiving anticoagulant therapy for venous thromboembolism: findings from the RIETE registry. J Thromb Haemost 8:1216–1222 20345727 10.1111/j.1538-7836.2010.03852.x

[6] Johnsen HS, Braekkan SK, Morelli VM, Hansen J-B (2021) Platelet count and risk of major bleeding in venous thromboembolism. Platelets 32:444–452 32498591 10.1080/09537104.2020.1769052

[7] Samuelson Bannow BT, Lee A, Khorana AA, Zwicker JI, Noble S, Ay C, Carrier M (2018) Management of cancer-associated thrombosis in patients with thrombocytopenia: guidance from the SSC of the ISTH. J Thromb Haemost 16:1246–1249. 10.1111/jth.14015 29737593 10.1111/jth.14015

[8] Galvez C, Stein BL (2020) Thrombocytosis and Thrombosis: Is There Really a Correlation? Curr Hematol Malig Rep 15:261–267. 10.1007/s11899-020-00588-z 32399765 10.1007/s11899-020-00588-z

[9] Schafer AI (2006) Molecular basis of the diagnosis and treatment of polycythemia vera and essential thrombocythemia. Blood 107:4214–4222 16484586 10.1182/blood-2005-08-3526

[10] Heestermans M, Poenou G, Duchez AC, Hamzeh-Cognasse H, Bertoletti L, Cognasse F (2022) Immunothrombosis and the Role of Platelets in Venous Thromboembolic Diseases. Int J Mol Sci 23. 10.3390/ijms232113176. 10.3390/ijms232113176 PMC9656618 36361963

[11] Zakai N, Wright J, Cushman M (2004) Risk factors for venous thrombosis in medical inpatients: validation of a thrombosis risk score. J Thromb Haemost 2:2156–2161 15613021 10.1111/j.1538-7836.2004.00991.x

[12] Simanek R, Vormittag R, Ay C, Alguel G, Dunkler D, Schwarzinger I, Steger G, Jaeger U, Zielinski C, Pabinger I (2009) High platelet count associated with venous thromboembolism in cancer patients: results from the Vienna Cancer and Thrombosis Study (CATS). J Thromb Haemost 8:114–120 19889150 10.1111/j.1538-7836.2009.03680.x

[13] Albertin CL, Uppal S, Al-Niaimi AN, Seo S, Hinshaw JL, Hartenbach EM (2015) Thrombocytosis is Predictive of Postoperative Pulmonary Embolism in Patients With Gynecologic Cancer. Int J Gynecol Cancer 25:1096–1101. 10.1097/igc.0000000000000462 26098091 10.1097/IGC.0000000000000462

[14] Ho K, Yip C, Duff O (2012) Reactive thrombocytosis and risk of subsequent venous thromboembolism: a cohort study. J Thromb Haemost 10:1768–1774 22784217 10.1111/j.1538-7836.2012.04846.x

[15] Kovács S, Csiki Z, Zsóri KS, Bereczky Z, Shemirani AH (2019) Characteristics of platelet count and size and diagnostic accuracy of mean platelet volume in patients with venous thromboembolism A systematic review and meta-analysis. Platelets 30:139–147 29252063 10.1080/09537104.2017.1414175

[16] Monreal M, Urrutia A, Marti S, Cuxart A, Roncales J (1997) Platelet count and the risk of bleeding in hospitalized patients with venous thromboembolism starting anticoagulant therapy. Haemostasis 27:91–98. 10.1159/000217439 9212357 10.1159/000217439

[17] Méan M, Righini M, Jaeger K, Beer H-J, Frauchiger B, Osterwalder J, Kucher N, Lämmle B, Cornuz J, Angelillo-Scherrer A (2013) The Swiss cohort of elderly patients with venous thromboembolism (SWITCO65+): rationale and methodology. J Thromb Thrombolysis 36:475–483 23359097 10.1007/s11239-013-0875-2

[18] Erkurt MA, Kaya E, Berber I, Koroglu M, Kuku I (2012) Thrombocytopenia in adults. *Journal of*. Hematology 1:44–53

[19] Schafer AI (2004) Thrombocytosis. N Engl J Med 350:1211–1219 15028825 10.1056/NEJMra035363

[20] Schulman S, Kearon C (2005) Definition of major bleeding in clinical investigations of antihemostatic medicinal products in non-surgical patients. J Thromb Haemost 3:692–694 15842354 10.1111/j.1538-7836.2005.01204.x

[21] Büller H, Gent M, Gallus A, Ginsberg J, Prins M, Baildon R, Investigators C (1997) Low-molecular-weight heparin in the treatment of patients with venous thromboembolism. N Engl J Med 337:657–662 9280815 10.1056/NEJM199709043371001

[22] Jakobsson C, Jimenez D, Gomez V, Zamarro C, Mean M, Aujesky D (2010) Validation of a clinical algorithm to identify low-risk patients with pulmonary embolism. J Thromb Haemost 8:1242–1247 20230422 10.1111/j.1538-7836.2010.03836.x

[23] Rosendaal FR, Cannegieter SC, van der Meer FJ, Briët E (1993) A method to determine the optimal intensity of oral anticoagulant therapy. Thromb Haemost 69:236–239 8470047

[24] Fine JP, Gray RJ (1999) A proportional hazards model for the subdistribution of a competing risk. J Am Stat Assoc 94:496–509

[25] Wells PS, Tritschler T, Khan F, Anderson DR, Kahn SR, Lazo-Langner A, Carrier M, Le Gal G, Castellucci LA, Shah V (2022) Predicting major bleeding during extended anticoagulation for unprovoked or weakly provoked venous thromboembolism. Blood Adv 6:4605–4616 35679460 10.1182/bloodadvances.2022007027 PMC9636329

[26] Ferrazzini E, Méan M, Stalder O, Limacher A, Rodondi N, Aujesky D (2023) Incidence and clinical impact of bleeding events in older patients with acute venous thromboembolism. Blood Adv 7:205–213 35381071 10.1182/bloodadvances.2022007263 PMC9841039

[27] Lauber S, Limacher A, Tritschler T, Stalder O, Mean M, Righini M, Aschwanden M, Beer JH, Frauchiger B, Osterwalder J, Kucher N, Lammle B, Cornuz J, Angelillo-Scherrer A, Matter CM, Husmann M, Banyai M, Staub D, Mazzolai L, Hugli O, Rodondi N, Aujesky D (2018) Predictors and Outcomes of Recurrent Venous Thromboembolism in Elderly Patients. Am J Med 131(703):e7–e16. 10.1016/j.amjmed.2017.12.015 29307536 10.1016/j.amjmed.2017.12.015

[28] Hansson P-O, Sörbo J, Eriksson H (2000) Recurrent venous thromboembolism after deep vein thrombosis: incidence and risk factors. Arch Intern Med 160:769–774 10737276 10.1001/archinte.160.6.769

[29] Faller N, Limacher A, Méan M, Righini M, Aschwanden M, Beer JH, Frauchiger B, Osterwalder J, Kucher N, Lämmle B (2017) Predictors and causes of long-term mortality in elderly patients with acute venous thromboembolism: a prospective cohort study. Am J Med 130:198–206 27742261 10.1016/j.amjmed.2016.09.008

[30] Trujillo-Santos J, Ruiz-Gamietea Á, Luque JM, Samperiz ÁL, Garcia-Bragado F, Todoli JA, Monreal M (2009) Predicting recurrences or major bleeding in women with cancer and venous thromboembolism. Findings from the RIETE Registry. Thromb Res 123:S10–S5. 10.1016/S0049-3848(09)70003-9 19217463 10.1016/S0049-3848(09)70003-9

[31] Monreal M, Falgá C, Valdés M, Suárez C, Gabriel F, Tolosa C, Montes J, Investigators R (2006) Fatal pulmonary embolism and fatal bleeding in cancer patients with venous thromboembolism: findings from the RIETE registry. J Thromb Haemost 4:1950–1956 16961602 10.1111/j.1538-7836.2006.02082.x

[32] Liebman HA (2014) Thrombocytopenia in cancer patients. Thromb Res 133:S63–S69 24862148 10.1016/S0049-3848(14)50011-4

[33] Toutenburg H (1990) Rubin, D.B.:Multiple imputation for nonresponse in surveys. Stat Pap 31:180. 10.1007/BF02924688

[34] Ruíz-Giménez N, Suárez C, González R, Nieto JA, Todolí JA, Samperiz ÁL, Monreal M, Investigators R (2008) Predictive variables for major bleeding events in patients presenting with documented acute venous thromboembolism. Findings from the RIETE Registry. Thromb Haemost. 100:26–31 18612534 10.1160/TH08-03-0193

[35] Frei AN, Stalder O, Limacher A, Méan M, Baumgartner C, Rodondi N, Aujesky D (2021) Comparison of bleeding risk scores in elderly patients receiving extended anticoagulation with vitamin K antagonists for venous thromboembolism. Thromb Haemost 121:1512–1522 33930905 10.1055/s-0041-1726345

[36] Falanga A, Leader A, Ambaglio C, Bagoly Z, Castaman G, Elalamy I, Lecumberri R, Niessner A, Pabinger I, Szmit S, Trinchero A, Ten Cate H, Rocca B (2022) EHA Guidelines on Management of Antithrombotic Treatments in Thrombocytopenic Patients With Cancer. Hemasphere 6:e750. 10.1097/hs9.0000000000000750 35924068 10.1097/HS9.0000000000000750 PMC9281983

[37] Samuelson Bannow BT, Walter RB, Gernsheimer TB, Garcia DA (2017) Patients treated for acute VTE during periods of treatment-related thrombocytopenia have high rates of recurrent thrombosis and transfusion-related adverse outcomes. J Thromb Thrombolysis 44:442–447. 10.1007/s11239-017-1539-4 28884390 10.1007/s11239-017-1539-4 PMC5659888

[38] Khanal N, Bociek RG, Chen B, Vose JM, Armitage JO, Bierman PJ, Maness LJ, Lunning MA, Gundabolu K, Bhatt VR (2016) Venous thromboembolism in patients with hematologic malignancy and thrombocytopenia. Am J Hematol 91:E468–E472. 10.1002/ajh.24526 27489982 10.1002/ajh.24526

[39] Khorana AA, Francis CW, Culakova E, Lyman GH (2005) Risk factors for chemotherapy-associated venous thromboembolism in a prospective observational study. Cancer: Interdisciplinary Int J Am Cancer Soc 104:2822–9 10.1002/cncr.21496 16284987

[40] Mandalà M, Barni S, Prins M, Labianca R, Tondini C, Russo L, Milesi A, Cremonesi M, Zaccanelli M, Regonesi C, Moro C, Falanga A (2010) Acquired and inherited risk factors for developing venous thromboembolism in cancer patients receiving adjuvant chemotherapy: a prospective trial. Ann Oncol 21:871–876. 10.1093/annonc/mdp354 19713246 10.1093/annonc/mdp354

[41] Napolitano M, Saccullo G, Marietta M, Carpenedo M, Castaman G, Cerchiara E, Chistolini A, Contino L, De Stefano V, Falanga A (2019) Platelet cut-off for anticoagulant therapy in thrombocytopenic patients with blood cancer and venous thromboembolism: an expert consensus. Blood Transfus 17:171 30418130 10.2450/2018.0143-18 PMC6596377

[42] Geldhof V, Vandenbriele C, Verhamme P, Vanassche T (2014) Venous thromboembolism in the elderly: efficacy and safety of non-VKA oral anticoagulants. Thromb J 12:21. 10.1186/1477-9560-12-21 25650285 10.1186/1477-9560-12-21 PMC4314657

